# Flexo-Ionic Effect of Ionic Liquid Crystal Elastomers

**DOI:** 10.3390/molecules26144234

**Published:** 2021-07-12

**Authors:** C. P. Hemantha Rajapaksha, M. D. Tharindupriya Gunathilaka, Suresh Narute, Hamad Albehaijan, Camilo Piedrahita, Pushpa Paudel, Chenrun Feng, Björn Lüssem, Thein Kyu, Antal Jákli

**Affiliations:** 1 Department of Physics, Kent State University, Kent, OH 44240, USA; crajapak@kent.edu (C.P.H.R.); ppaudel1@kent.edu (P.P.); blussem@kent.edu (B.L.); 2 Advanced Materials and Liquid Crystal Institute, Kent State University, Kent, OH 44240, USA; mtharindupri_gst@kent.edu (M.D.T.G.); cfeng4@kent.edu (C.F.); 3 Department of Polymer Engineering, University of Akron, Akron, OH 44325, USA; stn14@uakron.edu (S.N.); haa76@zips.uakron.edu (H.A.); cpiedrahita@zips.uakron.edu (C.P.); tkyu@uakron.edu (T.K.)

**Keywords:** ionic liquid crystal elastomers, ionic liquids, flexoelectricity, flexo-ionic effect, micropower generation, sensors, electromechanical coupling

## Abstract

The first study of the flexo-ionic effect, i.e., mechanical deformation-induced electric signal, of the recently discovered ionic liquid crystal elastomers (iLCEs) is reported. The measured flexo-ionic coefficients were found to strongly depend on the director alignment of the iLCE films and can be over 200 µC/m. This value is orders of magnitude higher than the flexo-electric coefficient found in insulating liquid crystals and is comparable to the well-developed ionic polymers (iEAPs). The shortest response times, i.e., the largest bandwidth of the flexo-ionic responses, is achieved in planar alignment, when the director is uniformly parallel to the substrates. These results render high potential for iLCE-based devices for applications in sensors and wearable micropower generators.

## 1. Introduction

The past few decades have witnessed a flourishing development of electro-mechanical transducers for use in soft robotics [1,2,3], sensors [4,5] and micropower generations [6,7,8,9]. Piezoelectricity and flexoelectricity are the two major physical mechanisms for linear electro-mechanical transduction for insulating materials. Piezoelectricity is restricted only to non-centrosymmetric materials and linearly couples mechanical strain and electric signals [10].

Flexoelectricity exists even in centrosymmetric materials by coupling electric polarization with strain gradient. It has been studied in both solid crystals and liquid crystals since the 1960s [11]. In crystalline solids, the induced electric polarization is attributed to the displacement of electrons. The highest flexo-electric coefficients in the range of 1–100 μC/m have been reported for ferro-electric ceramics [12,13], whereas in dielectric polymers, the values are several orders of magnitudes smaller, in the range of 10^−2^–10^−3^ μC/m [14,15]. In liquid crystals, the dipolar origin of the flexoelectricity arises when the director structure of dipolar pear (tear drop) or bent (banana) shape molecules suffer splay or bend distortions, respectively [11]. The flexo-electric coupling coefficients that arise from such dipolar contribution were measured to be very low, i.e., 1–10 pC/m [16,17]. Later, an effect three orders of magnitude larger (~60 nC/m) was observed in bent-core liquid crystals, but that was attributed to strain-gradient-induced reorientation of 10–100 nm scale ferro-electric smectic clusters [18]. Bent core nematic liquid crystal elastomers have also shown a large (~30 nC/m) flexo-electric coefficient [19]. They are much less brittle than rigid ceramics, and they are self-standing and rugged unlike fluid nematics.

In the past two decades, ionic electro-active polymers (iEAPs) have attracted widespread attention due to their unique properties that can be used in soft robotics as actuators [20,21,22,23,24] and sensors [24,25]. In these systems, due to the different sizes of the anions and cations, the applied voltage leads to a mechanical bending which itself facilitates the movement of larger ions toward the convex sign of the bent film. Such a mechanism provides low-voltage operation for actuators, and large-current response for sensors, thus making them attractive for various applications.

Recently, a new class of strain-gradient-induced electric current was demonstrated in ionic polymer membranes [9]. In appearance, this effect is similar to the flexo-electric effect. However, instead of the displacement of bound charges, here, positive and negative ions move in opposite directions due to mechanical bending [6,7,8,9,26]. To express this difference, we shall call this the flexo-ionic effect. Indeed, the flexo-ionic coefficients of these materials are much larger (29–323 μC/m) than those of the flexo-electric coefficients of insulating polymers. The flexo-ionic effect is the converse of the electro-actuation of ionic polymers and elastomers (iEAPs) containing different sizes of cations and anions [3,23,27,28,29,30,31,32,33,34,35,36].

Most recently, our group has demonstrated electro-actuation of a new class of materials, ionic liquid crystal elastomers [1]. We showed that iLCEs can be actuated by low frequency AC or DC voltages of less than 1 V with bending strains comparable to the well-developed iEAPs. It was also shown that the fastest and strongest electro-actuation is observed for films where the director is uniformly parallel to the substrates (planar alignment). This implies the possibility of a pre-programed actuation pattern at the stage of the cross-linking process, and dual (thermal and electric) actuations in hybrid samples.

Here, we report the study of the converse effect, i.e., the flexo-ionic effects of iLCEs. Similar to electro-actuation, we find a strong alignment dependence of the flexo-ionic effect that is comparable to those found in ionic polymer membranes.

## 2. Results and Discussion

**Figure 1:**
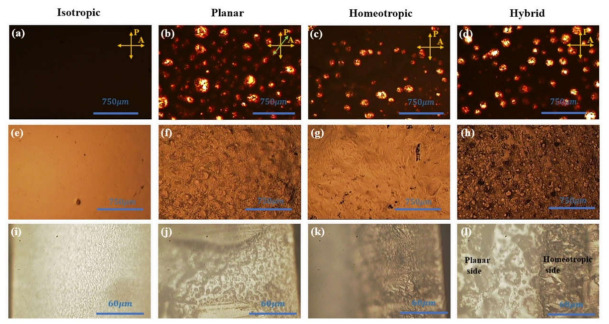
POM images of 200-µm thick iLCE samples cross-linked in the isotropic phase (first column, **a**,**e**,**i**), in the nematic phase between planar alignment substrates (second column, **b**,**f**,**j**), between homeotropic alignment substrates (third column, **c**,**g**,**k**), and in hybrid alignment between planar (top) and homeotropic (bottom) substrates (fourth column, **d**,**h**,**l**). First row (**a**–**d**): top view of iLCE samples between cross polarizers in transmission. Second row (**e**–**h**): top view of iLCEs in reflection mode. Third row (**i**–**j**): cross section of iLCEs in reflection (the substrates are on the left and right and the director in planar alignment points normal to the picture plane).

POM images of 200 µm thick iLCE samples prepared as described in the Materials and Method section and cross-linked in the isotropic phase, in the nematic phase between planar and homeotropic alignment substrates, and in hybrid alignment (between planar and homeotropic substrates) are shown in the 1st to 4th columns, respectively, from left to right in Figure 1. For the sake of brevity, these materials are referred to as isotropic, planar, homeotropic, and hybrid samples, respectively. The top row (a–d) shows the top view of iLCE samples between cross polarizers in transmission. As depicted in Figure 1a, the isotropic sample appeared to be completely dark, confirming the optical isotropy of the film. Bright droplets in the dark (isotropic) background in Figure 1b–d indicate spinodal decomposition of the isotropic ionic liquid-rich and the birefringent (bright) nematic LCE-rich phases [37]. The decomposition was induced by the isotropic-nematic transition and was found to strongly depend on the time the sample stayed in the nematic phase before the completion of the cross-linking reaction. After cross-linking, the phase separation was halted, and the size of the birefringent and isotropic domains did not change. For this reason, the cross-linking reaction was carried out at ~50 °C, only a few degrees below the I-N transition.

The middle row (e–h) of Figure 1 displays the top view of iLCE samples in reflection. The surface morphology of the isotropic film (e) is smooth, while the surface morphology of planar (f), homeotropic (g), and hybrid (h) show inhomogeneous structures due to the phase separation between ionic liquid and nematic liquid crystal-rich phases.

Even though, due to the phase separation, there is no significant difference in texture in the top views of the samples cross-linked in the nematic phase, there is a significant difference in the side views, as seen in the bottom row of Figure 1. Similar to the transmission images, the isotropic (i) sample is uniform, while the planar (j), homeotropic (k), and hybrid (l) samples are inhomogeneous. Notably, while the top views of the planar and the hybrid (with planar side on the top) samples are darker (less reflective) than those of the isotropic and the homeotropic cells, the side views of the planar and isotropic samples are brighter (more reflective) than the homeotropic cells. Additionally, the hybrid cells show a sharp contrast between bright texture close to the planar surface and darker texture near the homeotropic substrate. Since in the side view, the planar director points toward the picture, we conclude that the ionic channels that reflect light have larger cross sections in the plane normal to the LCE director. These show that, although the surface alignments are somewhat disturbed by the phase separation, the bulk alignments are clearly influenced by the surface alignments.

As expected, DSC measurements revealed that the glass transition temperatures (T_g_) for all samples are the same (T_g_ ~22 °C). This indicates that the plasticization of iLCE is independent of the director alignment and depends only on the composition of the polymer matrix.

The tensile stress–strain curves of the different iLCE strips are not linear, even at small strains, which indicates strain-induced director realignment and/or ionic channel reconfigurations. The slopes give Young’s moduli values in the range of 1–3 MPa that are several times smaller than typical values of the pure LCEs. This implies that the ion channels have weakened the elastomeric networks. The maximum applicable strain before rupturing depends on the alignment; the isotropic film can be strained up to ~ 50%, while the hybrid, homeotropic, and planar films rupture at ~ 25%, ~12%, and ~10% strain, respectively. The threshold for the rupture is likely related to the structure of defects and phase separation, which are largely absent in the isotropic film and differently aligned in the planar and homeotropic cells.

The DC ionic conductivities σ of the samples are calculated using the equation σ=L/RA, where L is the thickness of the film, R is the resistance, and A is the active area of the film. The ionic conductivities are found to be 3.3 mS/m, 2.5 mS/m, 0.18 mS/m, and 0.03 mS/m for the planar, isotropic, homeotropic, and hybrid alignment, respectively. The high ionic conductivities of planar and isotropic samples and low ionic conductivities of the homeotropic and hybrid samples are consistent with the reflection images that indicated that the ionic channels that reflect light have larger cross sections in the plane normal to the LCE director, which is along the film thickness in the planar cells. Additionally, the boundary interface seen in the middle of the cross section of the hybrid film appears to block the ion transportation through the film, resulting in the lowest ionic conductivity.

**Figure 2:**
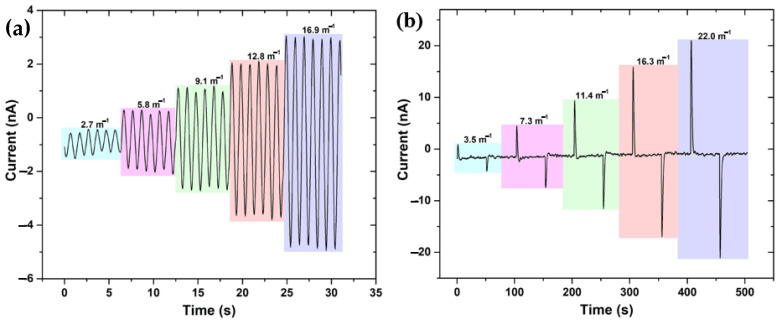
Flexo-ionic effect due to sinusoidal and intermittent cantilever bending (**a**) current response due to sine wave (1 Hz) bending with different curvatures (**b**) current response due to square wave (10 mHz) bending with different curvatures.

The measured time dependences of the electric currents in response to sinusoidal and square-wave (intermittent) cantilever bending, are depicted in Figure 2a,b, respectively. As shown in Figure 2a, the current response follows the sine wave with the same 1 Hz frequency. Figure 2b exhibits spikes during the intermittent bending following the direction of the intermittent bending with T = 100 s periodicity.

The bending-induced ionic current is due to the ion motion driven by the pressure gradient, which pushes the larger cations from the compressed (low volume), higher pressure side toward the expanded (high volume), low pressure side. Simultaneously, the smaller anions move in opposite directions. This leads to a charge separation that can be characterized by an effective dipole moment μ with a dimension of charge times distance, which is the net charge at the two substrates multiplied by the film thickness, μ=Q·d. The flexo-ionic polarization $P_{f-i}\;$, i.e., the dipole density, can be expressed as$\;P_{f-i}=\frac{\mu }{V}=Q/A$, where A is the electrode area. The net charge Q can be obtained by the time integral of the measured ionic current I as $Q=\int_{0}^{T}I\;dt,\;$where T, the period of the bending, is the inverse of the bending frequency, T=1/f. From these, we get the flexo-ionic polarization from the measured ionic current generated under the bending frequency f, as

(1) $$P_{f-i}=I/2\pi fA$$

**Figure 3:**
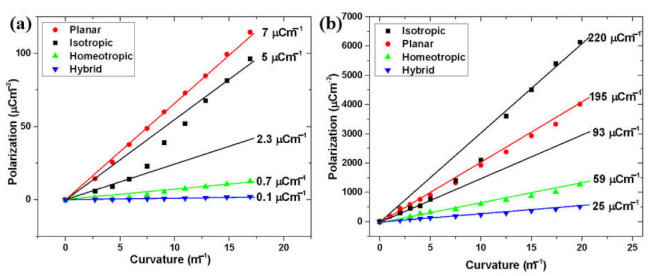
Flexo-ionic polarization versus bending curvature of differently aligned iLCE samples. (**a**) 1 Hz sine wave bending (**b**) 10 mHz square wave mechanical deformation.

The flexo-ionic polarization calculated by Equation (1) is plotted as the function of the curvature for sinusoidal and square-wave bending, which are shown in Figure 3a,b, respectively.

We see that the flexo-ionic polarization is basically proportional to the curvature for all samples that were cross-linked in the nematic phase, whereas the isotropic sample shows two linear regimes with a smaller slope at curvatures $\kappa <6{\;m}^{-1}$ and a larger slope at $\kappa >12{\;m}^{-1}.$ The slopes determine the flexo-ionic coupling constant, $e_{b}=P_{f-i}/\kappa$. In comparing Figure 3a,b, one can see that, for the 1 Hz sine wave, bending $e_{b}$ is largest ($e_{b}=7\;µC/m)$ for the planar sample and smallest ($e_{b}=0.1\;µC/m)\;$for the hybrid sample. On the other hand, for the 10 mHz square wave bending, the flexo-ionic coefficients are more than an order of magnitude larger. Similar to the 1 Hz sinusoidal bending, for $\kappa <6{\;m}^{-1}$ curvatures the largest coefficient is obtained in planar samples ($e_{b}=195\;µC/m$) followed by the isotropic ($e_{b}=93\;µC/m$), homeotropic ($e_{b}=59\;µC/m$), and hybrid ($e_{b}=25\;µC/m$) samples. At $\kappa >12{\;m}^{-1}$ curvatures, however, the isotropic sample overcomes the planar $e_{b}=220\;µC/m$ bending flexo-ionic coefficient.

**Figure 4:**
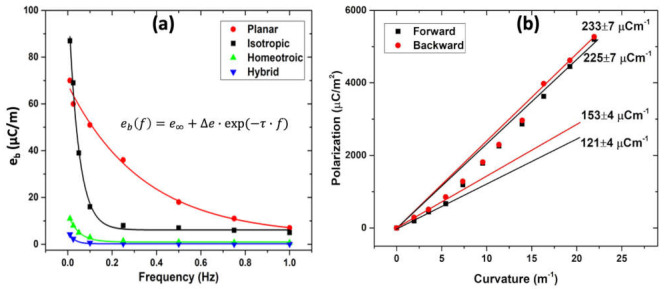
(**a**) Frequency dependence of the flexo-ionic coefficient of differently aligned iLCEs. Symbols are the experimental data points, and the solid lines are fitted curve to a single exponential function with the equation shown in the figure. (**b**) Flexo-ionic polarization versus increasing and decreasing curvature for the isotropic sample. The error values indicated in the figure are calculated by the standard deviation. Black solid squares denote the polarization measured during the curvature increment and solid red circles denote the polarization measured during the curvature decrement.

To find out if the large differences in the flexo-ionic coefficients were due to the difference in the time dependence of the bending (sine or square wave) or simply due to the difference in frequencies, we measured the frequency dependence of the flexo-ionic coefficients $e_{b}$ in sine wave excitation only. The results are plotted in Figure 4a. One can see that at very low frequencies (below 0.02 Hz), the isotropic sample has the largest response, but that decreases very quickly, and above 0.03 Hz, the planar cell overcomes all other alignments. The extrapolated $e_{b}$ at 10 mHz becomes comparable with the results obtained in 10 mHz rectangular voltages, indicating that the main factor responsible for the differences seen in Figure 3a,b is frequency and not the waveform. The best exponential fits for the frequency dependences of the time constant τ give 2.9 s for planar, 22.1 s for isotropic, 20.7 s for homeotropic, and 38.7 s for hybrid films. These values, within the experimental errors, are corroborated with the alignment dependences of the electric conductivity and are in agreement with those observed for the response times of the same materials in electro-actuation [1]. The non-zero $e_{\infty }$ values for the planar and isotropic samples indicate that a small portion of the sample reacts even faster (above 1 Hz).

To obtain an insight into the nature of the reproducibility, we have carried out the measurements both in increasing and decreasing curvatures for all materials. Within the error, no hysteresis has been observed for any of the samples, including the isotropic sample where the slope varies with the curvature. This latter one is shown in Figure 4b. The change of the slope at increasing curvature indicates a strain-gradient induced alignment of the mesogenic side chains from an isotropic distribution to an aligned structure. As the slope in the high bend region is closest to that of the planar sample, we conjecture that the bending-induced alignment is planar. The fact that in this range the isotropic strip shows an even larger flexo-ionic effect than the planar sample is probably due to the weaker phase separation and the lack of defects. The lack of hysteresis indicates that this realignment is completely reversible, i.e., the isotropic distribution of the director structure resumes reversibly.

## 3. Materials and Methods

### 3.1. Materials

**Figure 5:**
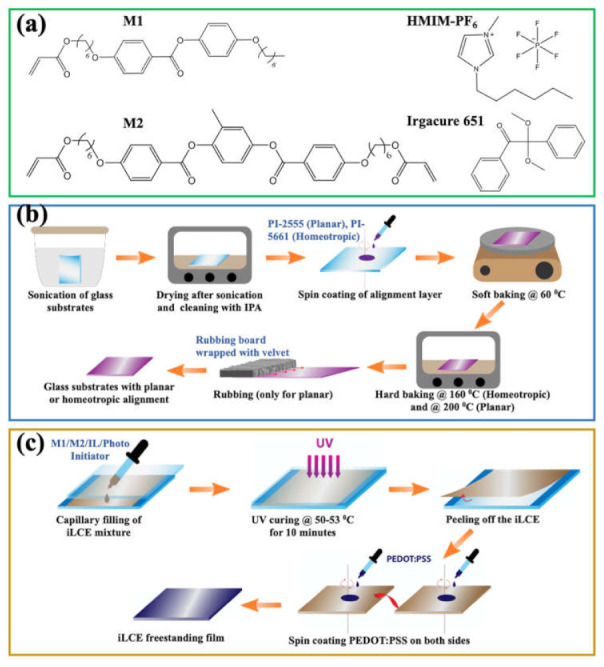
Molecular structures of the components of the iLCE materials and the illustration of sample preparation. (**a**) Molecular structures of the components of the studied iLCE: M1 and M2 are mesogenic units, HMIM-PF_6_ is the ionic liquid, and Irgacure 651 is the photoinitiator. (**b**) Schematic view of preparation of the planarly and homeotropically aligned glass substrates. (**c**) Schematic view of fabrication of differently aligned (planar, homeotropic, hybrid, isotropic) iLCE films with PEDOT:PSS electrodes.

Monofunctional acrylate monomer M1 (4-(6-Acryloxy-hex-1-yl-oxy) phenyl-4-(hexyloxy) benzoate) and bifunctional crosslinker M2 (1,4-Bis- [4-(6- acryloyloxyhexyloxy) benzoyloxy]-2-methylbenzene) were purchased from Synthon chemicals. IL (1-Hexyl-3-methylimidazolium hexafluorophosphate (HMIM-PF_6_)) and photoinitiator (2,2- Dimethoxy-2-phenylacetophenone (Irgacure^®^ 651)) were acquired from Sigma-Aldrich, Milwaukee, US. The molecular structures of these materials are illustrated in Figure 5a. For planar alignment polyimide PI-2555, and for homeotropic alignment PI-5661, alignment coatings were used as received from HD MicroSystems. For electrode materials, PEDOT:PSS (Clevios^TM^ PH1000) was mixed with 5% ethylene glycol (EG) and 0.25% dodecyl benzene sulfonic acid (DBSA) to enhance the conductivity [38].

### 3.2. Sample Preparations

M1, M2, and the photoinitiator were mixed in 87:12:1 weight ratio to form the LCE precursors. Subsequently, an ionic liquid (HMIM-PF_6_) was added to the LCE precursor solution and mechanically stirred for 15 min after heating to 80 °C to achieve complete mixing. A re-determined weight percentage (25%) of ionic liquid was used as per our previous experience on this system for electric actuation [1].

The substrate preparation and iLCE film fabrication are illustrated in Figure 5b,c. To prepare the planar-aligned and homeotropically aligned glass substrates (see Figure 5b), 25 mm by 25 mm glass pieces were sonicated for 20 min at 60 °C and washed with distilled water and isopropyl alcohol (IPA), followed by drying at 90 °C for 20 min. Afterward, a 10 nm thick layer of polyimide PI-2555 and PI-5661 was spin-coated on the glass substrates for planar and homeotropic alignments, respectively. After soft baking at 60 °C, the homeotropic alignment layer was hard baked at 160 °C for 1 h, and the planar alignment polyimide film was hard baked at 200 °C for 1 h and was rubbed unidirectionally with velvet cloth.

Four types of iLCE samples were prepared. One sample was cross-linked in the isotropic phase (called “Isotropic” sample) between two cleaned glass substrates with no alignment layer. Three were cross-linked in the nematic phase between planar alignment substrates (“Planar” sample), between homeotropic alignment substrates (“Homeotropic” sample), and in hybrid alignment between planar and homeotropic substrates (“Hybrid” sample). Figure 5c shows the steps to prepare 200 µm thick cells using capillary fill, UV irradiation for cross-linking, then peeling off the iLCE, coating both sides by PEDOT: PSS to achieve the freestanding films with electrodes.

The iLCE mixtures were filled to 200 µm cells at 80 °C (in the isotropic phase) by capillary action. Planar, homeotropic, and hybrid samples were photopolymerized under 365 nm UV light (Black-Ray, Model B-100AP/R) in the nematic phase (50–55 °C) for 10 min, while the isotropic sample was cross-linked in the isotropic phase (80 °C) under the same condition. Afterward, iLCE films were peeled off from the glass substrates, and about 100 nm thick PEDOT: PSS electrodes were spin-coated on both sides. Finally, the iLCE freestanding films with electrodes were cut into 2 mm by 15 mm pieces for material characterization and for the flexo-ionic measurements.

### 3.3. Experimental Techniques

The top views and the cross sections of iLCE samples were captured using a polarized optical microscope (POM) from Olympus (model: BX60) in transmission and reflection modes.

The glass transition temperatures (T_g_) of iLCE films with different director alignments were determined by differential scanning calorimetry (DSC) using Model Q200 from Thermal Analysis Instruments Inc., TA. Samples weighing 5–10 mg were hermetically sealed in aluminum pans by using a clamping machine. An empty aluminum pan was used for reference. Thermal scans were performed under nitrogen gas flow from −75 °C to 50 °C with a scan-rate of 10 °C/min.

The tensile properties were determined using a dynamic mechanical analyzer (DMA Q800, TA Instruments). The instrument was operated under tension mode with an initial strain rate of 1% at room temperature.

To investigate the ionic conductivities of the four types of iLCE samples, 50 mV DC and 1 mHz square wave voltages were applied across the iLCE films, and the electric current was measured.

**Figure 6:**
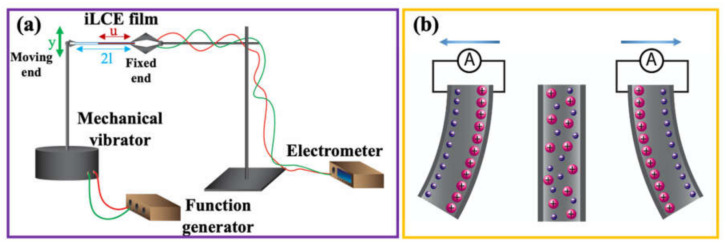
The schematic of the experimental setup, and the illustration of the physical mechanism of the flexo-ionic effect. (**a**) The schematic of the experimental setup to study flexo-ionic effect. (**b**) Illustration of the principle of the flexo-ionic effect.

The flexo-ionic effects of iLCE films were studied using a home-made setup as illustrated in Figure 6a. Sinusoidal and square wave oscillatory deformation were generated using a mechanical vibrator (SF-9324, PASCO) driven by a function generator (Agilent, 33120A). The ionic currents generated by the bending deformations were measured using an electrometer (6517B, Keithley) connected to a computer interface via GPIB (General Purpose Interface Bus) port by using a home-made LabView script. To determine the tip displacement y of the film, the images of bending were captured by a high-speed camera (Pixelink, 742000782) mounted to a microscope (Wild, M5-71977). A home-made Matlab (2019b) image processing script was used to analyze the images. The bending curvature κ of an iLCE film was calculated from the tip displacement y using the equation

(2) $$\kappa =\frac{1}{u}\frac{Slu-1/2\;Su^{2}}{\sqrt{1+{\left(Slu-1/2\;Su^{2}\right)}^{2}}}$$

where 2l is the distance between clamps, u is the active sample length, and $S=\frac{3y}{2l^{3}}$ [39].

The principle of the physical mechanism leading to the bending-induced ionic current is illustrated in Figure 6b.

## 4. Conclusions

We presented the first flexo-ionic effects of ionic liquid crystal elastomer samples in four different director configurations, isotropic, planar, homeotropic, and hybrid. We found that the measured flexo-ionic coupling constants strongly depend on the director structures and on the frequency of the mechanical bending. Morphology measurements carried out by POM and electric conductivity measurements show that the liquid crystal director structure and the phase of the liquid crystal where the cross-linking happens critically determines the structure and distribution of the ionic liquid channels. It appears that the cells cross-linked in the isotropic phase, and those that are cross-linked in the nematic phase between planar alignment substrates, give the largest response with flexo-ionic coefficients ~200 μC/m. Such coefficients are comparable to the well-developed iEAPs (see Table 1) [6,9]. It is interesting to discern that the flexo-ionic coefficients of iLCEs outperform the flexoelectric (polarization mechanism is different) coefficients of ceramics (PZT [12] and Ba_0.67_Sr_0.33_TiO_3_ [40]), crystalline polymers (PVDF) [41], and bent-core liquid crystals [18,19] as indicated in Table 1. This finding shows that at very low frequencies, the bending-induced charge separation of differently sized cations and anions of ionic liquids dispersed in polymers and liquid crystal elastomers are much more effective in leading to polarization than to separation of bound charges in insulating media. Our results, therefore, imply the possible use of iLCEs in sensors and energy harvesting devices where the mechanical excitations are slow (below 0.1 Hz). Further, ILCEs without spinodal decomposition are under investigation for the application of soft actuators, sensors, and energy harvesting devices.

## Figures and Tables

**Table 1:** Comparison of flexo-ionic coefficient and limit of the driving frequencies of iLCEs with ionic electroactive polymers, bent-core liquid crystals, and insulating polymers and ceramics.

Materials	Flexoelectric/Ionic Coefficient	Driving Frequencies
iLCEs (This study)	25–220 μC/m	0.01–1 Hz
PEM (TS-PEGDA/IL)	84–154 μC/m [6]	0.01–10 Hz
PEM (PEGDA/IL)	53–125 μC/m [6]	0.01–1 Hz
PEM (PEGDA/SCN/LiTFSI)	29–323 μC/m [9]	0.01 Hz
bent-core liquid crystal	60 nC/m [18]	1–10 Hz
Bent-core liquid crystal elastomer	30 nC/m [19]	0.2–12 Hz
PVDF	2–13 nC/m [41]	6 Hz
Pb [Zr_x_Ti_1−x_] O_3_ (PZT)	1.4 μC/m [12]	1 Hz
Ba_0.67_Sr_0.33_TiO_3_	100 μC/m [40]	2 Hz

## Data Availability

The data presented in this study are available on request from the corresponding author.

## Sample Availability

Samples of the compounds are not available from the authors.
